# The many facets of homologous recombination at telomeres

**DOI:** 10.15698/mic2015.09.224

**Published:** 2015-07-30

**Authors:** Clémence Claussin, Michael Chang

**Affiliations:** 1 European Research Institute for the Biology of Ageing, University of Groningen, University Medical Center Groningen, Groningen, The Netherlands.

**Keywords:** homologous recombination, telomeres, alternative lengthening of telomeres, telomerase-independent telomere maintenance, break-induced replication

## Abstract

The ends of linear chromosomes are capped by nucleoprotein structures called telomeres. A dysfunctional telomere may resemble a DNA double-strand break (DSB), which is a severe form of DNA damage. The presence of one DSB is sufficient to drive cell cycle arrest and cell death. Therefore cells have evolved mechanisms to repair DSBs such as homologous recombination (HR). HR-mediated repair of telomeres can lead to genome instability, a hallmark of cancer cells, which is why such repair is normally inhibited. However, some HR-mediated processes are required for proper telomere function. The need for some recombination activities at telomeres but not others necessitates careful and complex regulation, defects in which can lead to catastrophic consequences. Furthermore, some cell types can maintain telomeres via telomerase-independent, recombination-mediated mechanisms. In humans, these mechanisms are called alternative lengthening of telomeres (ALT) and are used in a subset of human cancer cells. In this review, we summarize the different recombination activities occurring at telomeres and discuss how they are regulated. Much of the current knowledge is derived from work using yeast models, which is the focus of this review, but relevant studies in mammals are also included.

## INTRODUCTION

Telomeres, nucleoprotein structures located at the ends of linear chromosomes, prevent natural chromosome ends from being recognized as DNA double-strand breaks (DSBs) (reviewed in 1). Telomere dysfunction can lead to inappropriate repair activities, such as homologous recombination (HR) and non-homologous end joining (NHEJ). Such activities at telomeres can result in chromosomal rearrangements and genomic instability. Due to incomplete DNA replication and nucleolytic degradation, telomeres shorten with each round of replication, eventually leading to a growth arrest, known as replicative senescence, or to apoptosis. Telomere shortening can however be counteracted by a specialized reverse transcriptase called telomerase, which is composed of a protein catalytic subunit and an RNA subunit 2,3,4,5. Telomerase extends telomeres by iterative reverse transcription of a short sequence to the 3' ends of telomeres, using the RNA subunit as a template 4,6,7.

Most human somatic cells do not express sufficient telomerase to prevent telomere shortening, which may be a contributing factor towards human ageing. This absence of telomere maintenance may have evolved as a barrier to tumorigenesis (reviewed in 8). Indeed, cancer cells need to activate a telomere maintenance mechanism (TMM), and in approximately 85-90% of cancers this occurs through the upregulation of telomerase 9. The remaining 10-15% of cancers employ telomerase-independent, recombination-based mechanisms, collectively termed alternative lengthening of telomeres (ALT) 10. ALT mechanisms were first described as a TMM in the budding yeast *Saccharomyces cerevisiae*, where these cells are called “survivors” 11. While recombination is clearly important for ALT and in survivors, recombination proteins are also important in non-ALT/survivor cells. For example, in *S. cerevisiae*, the combined absence of recombination and telomerase leads to a drastically enhanced rate of replicative senescence even though the rate of telomere shortening is apparently unchanged 11,12, although rare telomere loss events may be occurring. Furthermore, recombination proteins are important to resolve recombination intermediates at telomeres in pre-senescent cells 13, and can be detected at telomeres well before the appearance of survivors 14.

In this review, we will discuss how recombination is regulated at telomeres in telomerase-positive cells, telomerase-negative senescing cells, and telomerase-negative cells using recombination-mediated TMMs. Our focus will be on the significant advances made using different yeast models, but when appropriate, we will discuss relevant studies in mammalian systems.

## SUPPRESSION OF HOMOLOGOUS RECOMBINATION AT TELOMERES

**Figure 1:**
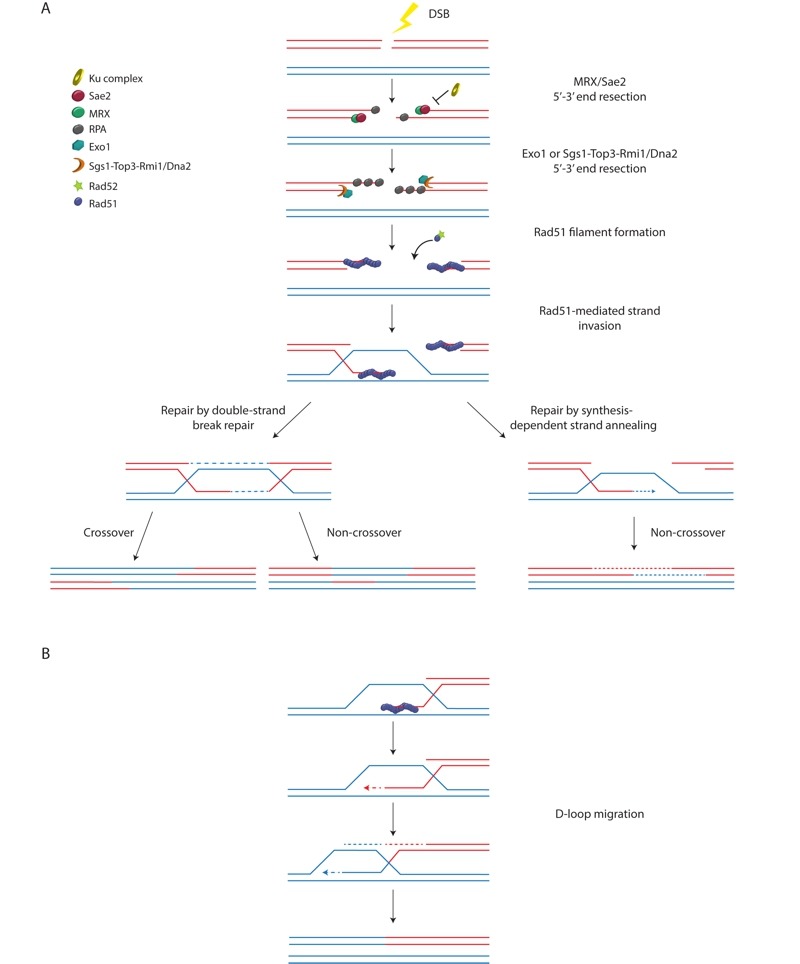
FIGURE 1: Models for homology-dependent DSB repair. **(A)** HR-mediated repair of a DSB is initiated by 5' to 3' resection of the DNA ends by the MRX complex and Sae2, and this resection is inhibited by the Ku complex. More extensive resection is then carried out by either Exo1 or the combined activities of the Sgs1-Top3-Rmi1 complex and Dna2. The resulting single-stranded DNA recruits the ssDNA-binding protein RPA. Rad52 mediates the loading of Rad51 onto RPA-coated ssDNA to form Rad51 nucleoprotein filaments capable of performing strand invasion. Repair can then proceed via the classical double-strand break repair model or the synthesis-dependent strand annealing model. **(B)** One-ended breaks can be repaired by BIR, which can be Rad51-dependent (as shown here) or Rad51-independent. In BIR, strand invasion leads to the formation of a D-loop that migrates along the chromosome as the invading 3' overhang is extended. The complementary strand is synthesized by conservative replication. For simplicity, not all proteins involved in DSB repair are shown.

**Figure 2:**
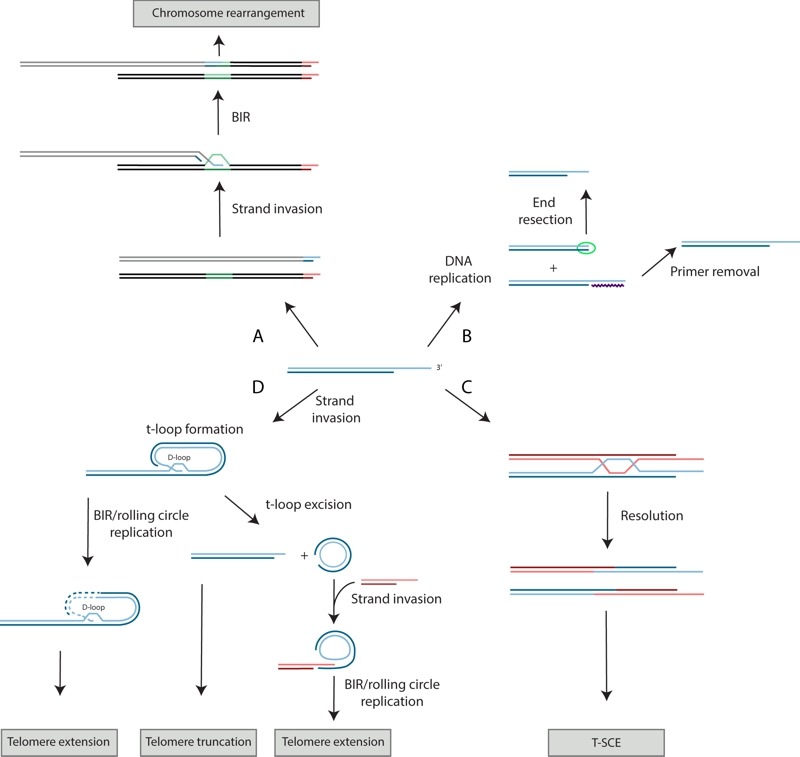
FIGURE 2: Recombination activities mediate a variety of telomere processes. **(A)** Strand invasion of a telomere into interstitial telomere sequence (green) located on the same (not shown) or different chromosome can lead to genome rearrangement. Depicted here, a segment of the invaded chromosome (black) is duplicated. **(B)** Replication of a telomere leads to two sister telomeres. The one synthesized by the lagging strand replication machinery will have an RNA primer (purple zigzag line) at its 5' terminus, while the other will have a blunt end (circled in green). Removal of the RNA primer on the former will lead to the regeneration of a 3' overhang while the latter must be 5' end resected. **(C)** A model for a T-SCE event. The blue and red lines depict sister telomeres. **(D)** A t-loop forms via the strand invasion of the telomeric 3' single-stranded overhang into double-stranded telomeric DNA of the same telomere. Excision of a t-loop yields a truncated telomere. Rolling circle DNA replication can be used to extend a telomere in a t-loop configuration, or a telomere (red) that has strand invaded a DNA circle containing telomeric repeats. Dashed lines indicate newly synthesize DNA.

HR can be defined as the exchange of DNA sequences between two homologous DNA molecules and can be used to repair DNA damage, in particular DSBs. Although there are multiple variations regarding how HR can be used to repair a DSB, all of these models initiate with the resection of the 5' ends of the break to yield 3' single-stranded tails, of which one, or both, can invade homologous double-stranded DNA and prime DNA synthesis, templated by the donor double-stranded DNA (reviewed in 15). These recombination intermediates are then processed by either helicases or resolvases, or both, to yield the final repaired product (Figure 1A). Ideally, both ends of a DSB remain in close proximity, but if this cannot be realized, a single end of a DSB can be repaired by an HR-mediated pathway termed break-induced replication (BIR). One-ended DSBs can also occur after the collapse of a replication fork. In BIR, the one-ended DSB invades a homologous sequence and replicates to the end of the invaded chromosome (Figure 1B). Since a BIR event could potentially result in extensive loss-of-heterozygosity, the BIR pathway is suppressed if both ends of a DSB are present 16. Although a telomere resembles a resected one-ended DSB, there is no evidence that BIR is constitutively active in non-ALT/survivor cells, suggesting that BIR must also be suppressed at functional telomeres. This suppression may stem from a need to prevent telomeres from recombining with chromosome-internal telomeric sequences, as such events would lead to chromosomal rearrangements, and potentially to gene duplications (Figure 2A). Alternatively, the suppression of BIR may function to prevent inappropriate ALT/survivor-like telomere lengthening.

It is difficult to accurately measure telomere recombination events, due in part to the uniformity of telomeric repeats. Such events, however, can be detected in *S. cerevisiae*. This is possible because *S. cerevisiae* telomeres consist of imperfect, degenerate repeats 17,18, which is caused by telomerase only using a portion of the RNA template in each extension cycle, and because the RNA template and telomeric DNA can align in different registers 19. Sequencing multiple copies of the same telomere derived from a clonal population of cells reveals a centromere-proximal region of stable sequence and a distal region with differing degenerate repeats 18,20. This degenerate distal region is largely abolished in the absence of telomerase 20, but rare sequence divergence events can be detected 21. Presumably, such events are occurring in the presence of telomerase as well, but it is possible that telomerase can influence recombination activity. These telomerase-independent events are thought to be due to unequal sister chromatid exchange or intertelomeric recombination, and occur at a frequency of less than 0.3% per telomere per generation 21. We have recently conducted more careful measurements indicating that the frequency of these events may even be substantially lower than 0.3% (C. Claussin and M. Chang, unpublished data), suggesting that while recombination proteins are important during senescence, as mentioned above, their activity does not result in unequal sister chromatid exchange and intertelomeric recombination events, as measured in this assay. Thus these events are normally tightly repressed until the emergence of survivors, when such events can readily be detected 22.

**Figure 3:**
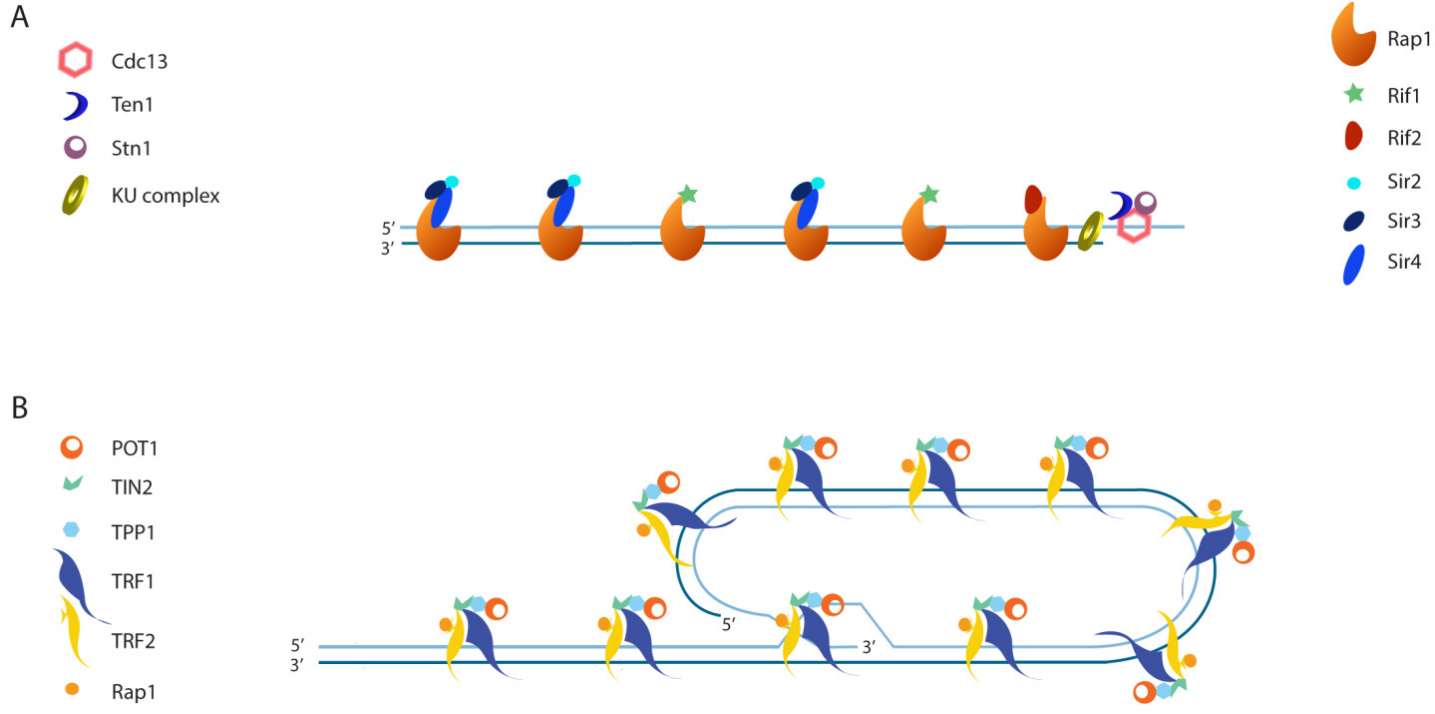
FIGURE 3: **(A)** Schematic of an S. cerevisiae telomere with associated proteins. **(B)** Schematic of shelterin-bound mammalian telomeres shown in a t-loop configuration.

The mechanism by which recombination of telomeric repeats is suppressed in *S. cerevisiae* is not entirely clear. Proteins that are present at a telomere but not at a DSB make obvious candidates to mediate the suppression (Figure 3A). The double-stranded portion of the telomere is bound by Rap1 23, which recruits the additional factors Rif1 and Rif2 24,25, as well as the silent chromatin proteins, Sir3 and Sir4 26. The CST complex (consisting of Cdc13, Stn1 and Ten1) binds to the 3' overhang 27,28,29,30,31.

Using the telomere sequencing approach described above, one study reported that the deletion of *RIF1* may increase telomeric recombination events, particularly at telomeres less than 120 bp in length 21, but a subsequent study failed to confirm this finding 22. Neither study found any role of Rif2 in suppressing recombination. A separate genetic assay designed to detect telomeric recombination events also found no role of Rif1, Rif2, or the Sir proteins in these events 32. In contrast, *cdc13-1* and* stn1-13* mutant strains exhibit elevated levels of telomeric recombination 30,32,33. The Ku heterodimer (consisting of Yku70 and Yku80) has also been shown to inhibit recombination at telomeres 32,34. Ku functions at both DSBs and telomeres to inhibit 5'-3' end resection, and accordingly, cells lacking Ku have increased 3' telomeric overhangs 34,35,36,37. The *cdc13-1* and *stn1-13* mutations also result in extensive telomere resection and long 3' overhangs 29,33. Since end resection is the first step in the processing of a DSB for subsequent recombination, these findings suggest that the CST and Ku complexes suppress telomeric recombination by inhibiting end resection at telomeres. As *cdc13-1 yku*∆ double mutants exhibit synthetic growth defects 34,38 and senesce after ~25 generations 32, it is likely that Cdc13 and the Ku complex function in separate pathways to inhibit resection. Consistent with this idea, Ku is more important for inhibiting resection in G1 and CST is more important in the S/G2 phases of the cell cycle 39.

While excessive telomeric resection is detrimental, some resection is needed to generate a 3' overhang (Figure 2B), which is needed for proper telomere capping. The 3' overhang is also the substrate for telomerase activity 40. In *S. cerevisiae*, end resection is initiated by the conserved MRX/N complex (Mre11, Rad50, and Xrs2 in *S. cerevisiae*, Nbs1 in other organisms), which together with Sae2 (CtIP in other organisms) can perform limited end resection 41,42. Following this initial step, more extensive resection is carried out by the 5'-3' exonuclease, Exo1, or the combined activities of the Sgs1-Top3-Rmi1 complex and Dna2 41,42 (Figure 1A). The Ku and CST complexes inhibit this more extensive resection. Rap1 and its associated protein Rif2 are also important for limiting MRX-mediated telomeric resection 37.

Similar to *S. cerevisiae*, telomeric recombination can be induced by perturbing telomere capping proteins in *Kluyveromyces lactis*. A mutation in the gene encoding *K. lactis* Stn1 (*stn1-M1*) causes survivor-like recombination-mediated telomere elongation, even in the presence of functional telomerase 43. Furthermore, telomerase-negative *K. lactis* mutants containing mutant repeats that disrupt Rap1 binding develop even longer telomeres than if they would have wild-type repeats, suggesting that Rap1 also plays a role in repressing recombination 44.

In addition to recombination of the telomeric tracts, the Ku complex also suppresses subtelomeric recombination in both *S. cerevisiae* 45 and *Schizosaccharomyces pombe* 46, although it is unclear how similar the mechanisms governing telomeric and subtelomeric recombination are. *S. pombe* cells lacking Taz1 (ortholog of mammalian shelterin components, TRF1 and TRF2; see below), which binds to the double-stranded portion of fission yeast telomeres, also exhibit elevated levels of subtelomeric recombination, which is thought to be a response to increased replication fork stalling 47.

In mammals, telomeric recombination, as measured by telomere sister chromatid exchange events (T-SCEs; Figure 2C), is inhibited by the combined action of POT1, TRF2, RAP1, and KU. Like Cdc13, POT1 binds to single-stranded telomeric DNA. Unlike yeast Rap1, human RAP1 has relatively weak DNA binding activity 48 and is instead recruited to telomeres by TRF2, which binds to double-stranded telomeric DNA. POT1, TRF2, and RAP1, along with TRF1, TIN2, and TPP1, form a six-membered telomere-capping complex called shelterin (Figure 3B; mouse shelterin has two POT1 orthologs (POT1a and POT1b) due to a recent duplication within the rodent lineage; reviewed in 1). Although loss of TRF2 or KU alone, or loss of both POT1a and POT1b together, exhibit only basal levels of T-SCEs (~1.5-3% of telomeres) in mouse embryonic fibroblasts (MEFs), T-SCEs are seen at approximately 10-15% of telomeres in MEFs lacking both TRF2 and KU, or in triple knockouts lacking POT1a, POT1b, and KU 49,50. The role of TRF2 in suppressing T-SCEs may be mediated by its recruitment of RAP1, as KU-deficient MEFs expressing an allele of TRF2 that cannot bind to RAP1 also exhibit elevated T-SCE levels 51.

## SOME RECOMBINATIONAL ACTIVITIES AT TELOMERES ARE BENEFICIAL

Although full activation of HR pathways must be prevented at telomeres, some recombination processes appear to be required for proper telomere function. In mammals, the 3' telomeric overhang can be further protected within a t-loop configuration (Figure 2D and Figure 3). A t-loop is a lariat structure formed by the invasion of the 3' overhang into the double-stranded portion of the same telomere. TRF2 is required for the formation and/or maintenance of t-loops 52,53. HR factors may be needed for the strand invasion step, as RAD51 and its paralog XRCC3, along with RAD52, can be detected at telomeres after replication, and these proteins are required for the generation of telomeric D-loops in an *in vitro* assay 54. However, recent biochemical studies indicate that TRF2 actually inhibits RAD51-mediated D-loop formation 55, and it also recruits the helicase RTEL1 to promote t-loop unwinding in S phase 56, indicating that TRF2 is both a positive and a negative regulator of t-loops. It has been proposed that the t-loop is important for disguising the chromosome ends, preventing the activation of the ATM checkpoint kinase and NHEJ 57, although it is possible that TRF2 directly inhibits ATM independently of t-loop formation 58. However, a t-loop also resembles an HR intermediate, which could lead to the formation of a Holliday junction, and thus has the potential to be excised through the action of resolvases, resulting in rapid telomere shortening (Figure 2D). Such excisions can occur in TRF2 mutants lacking its N-terminal basic domain (TRF2^∆B^) through a process that requires XRCC3 59. XRCC3 and RAD51C, both of whom are RAD51 paralogs, form a complex that is associated with Holliday junction resolvase activity *in vitro* 60. Furthermore, the N-terminal basic domain was recently found to inhibit GEN1 and MUS81, two endonucleases with resolvase activity 61. Thus, TRF2 is important for both the formation of t-loops and for preventing their excision from telomeres.

Unlike mammalian t-loops, yeast t-loops have been difficult to observe by electron microscopy because yeast telomeres are short, and long telomere restriction fragments, which can be separated from short genomic restriction fragments, are required for the visualization of native telomeric DNA. However, t-loops have been observed in *K. lactis* strains with elongated telomeres 62, and extrachromosomal DNA circles containing telomeric repeats, hypothesized to be excised t-loops, can be found in certain cell types, such as *S. cerevisiae* survivors 63,64.

While t-loop excision is normally repressed, over-elongated telomeres are specifically targeted for shortening by a mechanism called telomere rapid deletion (TRD), which is thought to occur through the excision of t-loops 65. TRD, also referred to as telomere trimming 66, was first seen in *S. cerevisiae*, where it was demonstrated that an over-elongated telomere can be shortened to wild-type length via a t-loop-excising intrachromosomal recombination event 67,68. TRD has been observed in *K. lactis* 69, *Arabidopsis thalania* 70, and human cells 66,71. Whether over-elongated telomeres are actively targeted for shortening by TRD is unclear, and the mechanism by which short or wild-type length telomeres, but not over-elongated telomeres, are protected from TRD has yet to be elucidated.

Proteins involved in HR are also required for proper replication of telomeres. Recombination processes are important for dealing with stalled or collapsed replication forks (reviewed in 72), and telomere sequences are known to cause problems for the replication machinery in *S. cerevisiae* 73, *S. pombe* 74, and mammals 75. Mouse cells lacking BRCA2, RAD51, RAD51C, RAD51D, and RAD54 have short telomeres and show signs of telomere fragility 76,77,78.

One reason that telomeres are difficult to replicate may be due to the transcription of telomeres, which produces long non-coding RNA called TERRA (telomeric repeat containing RNA). Co-transcriptionally-formed RNA-DNA hybrids (also referred to as R-loops) or the RNA polymerase II machinery itself can hinder DNA replication fork progression, which can lead to transcription-associated recombination (reviewed in 79). In* S. cerevisiae*, increasing the rate of telomere transcription induces Exo1-mediated telomere resection, which promotes telomeric recombination 80. Likewise, TERRA RNA-DNA hybrids, which can be resolved by RNase H and the THO complex (named after one of its subunits, Tho2), can also induce recombination. Mutating either RNase H or the THO complex increases the abundance of RNA-DNA hybrids at telomeres, leading to an increase in telomeric recombination 81,82,83. Thus, both the process of transcribing telomeres and TERRA R-loops can independently lead to telomeric recombination, which may be needed to preserve telomere integrity, especially in the absence of telomerase 80.

## TELOMERE MAINTENANCE VIA RECOMBINATION

**Figure 4:**
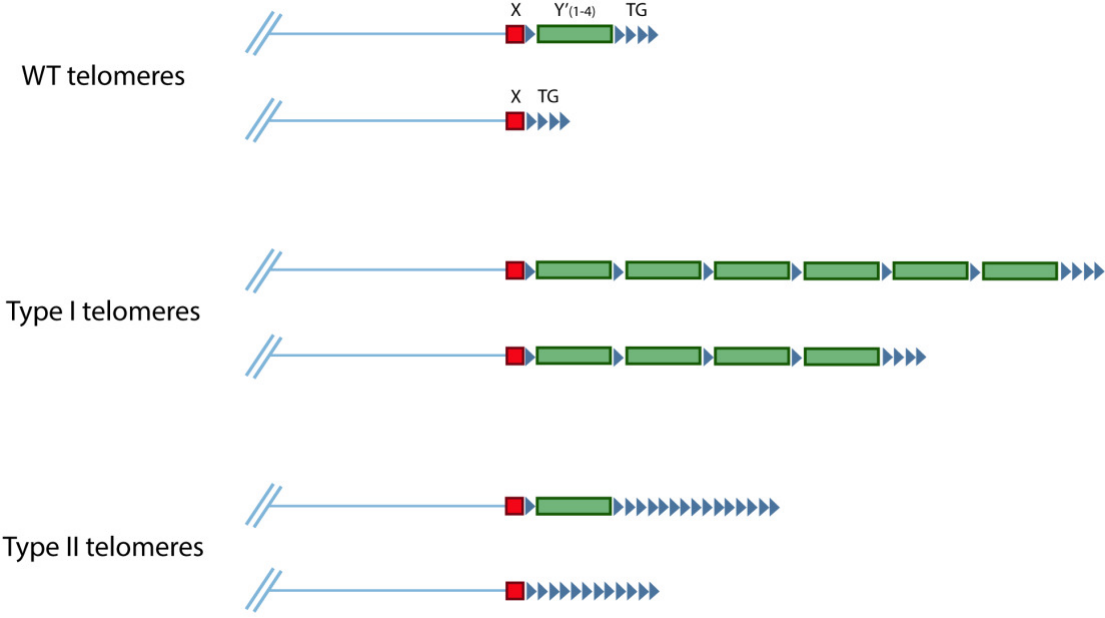
FIGURE 4: Schematic of *S. cerevisiae* telomeres in wild-type telomerase-positive cells (top), type I survivors (middle), and type II survivors (bottom). All wild-type telomeres contain an X element, and approximately half to two-thirds also contain one to four Y' elements. In type I survivors, Y' elements are amplified, even in telomeres that did not originally have a Y' element. The terminal telomeric repeats are amplified in type II survivors.

In the absence of telomerase, telomeres shorten until they activate the DNA damage checkpoint, which in turn stops further cell proliferation. However, some cells can overcome this barrier by using recombination-mediated mechanisms to elongate their telomeres. Such cells were first discovered in *S. cerevisiae*, where they are called survivors 11. There are two main types of survivors: type I survivors exhibit amplification of Y' subtelomeric elements, while type II survivors exhibit amplification of the terminal telomeric repeats (Figure 4) 11,84. Both types require Rad52, needed for almost all recombination in *S. cerevisiae*, and Pol32, a non-essential subunit of DNA polymerase δ that is required for BIR 11,85. The importance of Pol32 indicates that, in the absence of telomerase, BIR-mediated mechanisms can maintain telomeres, and that the suppression of BIR at telomeres must be alleviated in survivors. Deletion of *PIF1* also greatly impairs the formation of both types of survivors 86,87, which is likely attributable to the role of Pif1 in BIR 88. BIR can take place in a Rad51-dependent manner, or in a Rad51-independent manner that requires the MRX complex and Rad59 89,90. In addition to Rad52 and Pol32, type I survivors require Rad51, Rad54, and Rad57, whereas type II survivors require the MRX complex and Rad59 instead 12,91. This strongly suggests that telomere maintenance in type I survivors involve Rad51-dependent BIR while Rad51-independent BIR is important for type II survivors.

Sgs1 and Exo1 are also important for type II survivor formation 92,93,94,95. Sgs1 and Exo1 are needed for processive resection of DNA ends 41,42, suggesting that end resection might promote type II survivor formation by helping to generate a 3' overhang to initiate BIR. Consistent with this idea, an *sgs1-D664∆ *mutation, which is still competent in recombination repair but defective in resection 96,97, also prevents type II survivor formation 98. Interestingly, extensive resection by Sgs1 and Exo1 inhibits Rad51-dependent BIR 99,100. Thus, deletion of *SGS1* or *EXO1* may both promote the Rad51-dependent type I pathway and disrupt the Rad51-independent type II pathway. Similarly, deletion of *FUN30*, which encodes a chromatin remodeler that promotes end resection, partially hinders the formation of type II survivors 101.

A number of additional proteins have been implicated in the generation of type II survivors. These include the DNA damage checkpoint kinases Mec1 and Tel1 102, the B-type cyclin Clb2 103, Def2, an RNA polymerase II degradation factor, 104, Mdt4/Pin4, a protein that interacts with the checkpoint kinase Rad53 105, and Sua5, a protein required for an essential tRNA modification 106. A recent screen identified a further 22 genes important for type II survivor formation, including genes encoding for members of the KEOPS complex, the Rad6 DNA repair pathway, and proteins involved in nonsense-mediated decay 87. The same screen also identified that the INO80 chromatin remodeling complex affects the emergence of type I survivors 87. Exactly how these genes affect survivor formation is unclear.

Telomere length just before the emergence of survivors has also been shown to affect the ratio of type I to type II survivors formed, with long telomeres favoring type II survivors 22. Deletion of *RIF1* and *RIF2* strongly biases toward type II survivor formation 107, which is likely due to telomerase-negative *rif* mutants senescing with longer telomeres than telomerase-negative *RIF* strains 22. However, it is also possible that the Rif proteins limit type II survivor formation by inhibiting end resection 37.

Type I survivors typically arise more frequently but grow very poorly, whereas the growth of type II survivors is comparable to telomerase-positive cells 11,84. Rad51-dependent BIR is more efficient than Rad51-independent BIR 16,89, which may explain the higher frequency of type I survivors. The poor growth of type I survivors may be due to the maintenance of very short telomeres in these cells. Like senescent cells, type I survivors are arrested at the G2/M boundary with telomeres moving back and forth between the mother and the bud 108, indicating that the telomeres of type I survivors do not return to a properly capped state. In contrast, the long, heterogeneously sized telomeres of type II survivors behave in this respect like telomeres in telomerase-positive cells 108.

In type I survivors, all telomeres are extended through the amplification of subtelomeric Y' elements, even telomeres that did not originally have Y' elements 11. The movement of Y' sequences among chromosome ends can be explained as a BIR event initiated by an uncapped telomere that invades TG_1-3_ repeats that are found between some tandem Y' elements, or between some X and Y' elements (Figure 4) 109. While the formation of type I survivors is Rad51-dependent, efficient movement of Y' elements in the senescing phase before the emergence of survivors is facilitated more so by Rad59 than Rad51 110, which is surprising given that Rad59 is not required for type I survivor formation. The Y' element encodes a poorly characterized helicase that is strongly induced in type I survivors and this helicase may be important for the viability of type I survivors 111. Thus, amplification of the Y' elements may facilitate this process, but it is also possible that Y' amplification is only needed to provide homologous sequences at every telomere to allow for more efficient BIR. It is also unclear how the short terminal TG_1-3_ sequences are maintained.

Type II survivors are thought to elongate telomeres through a ‘roll-and-spread’ mechanism, involving both rolling circle synthesis and intertelomeric BIR events 112. Support for such a model is largely based on studies from *K. lactis*, where all survivors are type II due to a lack of subtelomeric blocks of telomeric repeats to allow for a type I-like pathway 113. *K. lactis* survivors derived from cells with two kinds of telomere repeats (i.e. repeats that are either wild type or mutant in sequence) usually contain repeating patterns in the lengthened telomeres, most likely arising from small circles containing telomere DNA being used as templates for rolling circle replication 114. Furthermore, transformation of a DNA circle containing mutant telomere repeats into a *K. lactis* telomerase-negative strain results in the incorporation of long tandem arrays of the mutant repeats at telomeres 114. These observations led to a model whereby a circle containing telomeric DNA (i.e. a t-circle) is produced by a recombination event, possibly through the excision of a t-loop 62. An uncapped telomere can then initiate BIR-mediated rolling circle DNA synthesis using the t-circle as a template (Figure 2D). In addition to being observed in *K. lactis* 115, t-circles are also found in *S. cerevisiae* survivors 63 64 and human ALT cells 59 116.

*S. pombe* can also form telomerase-negative survivors either by circularizing their three chromosomes (in a process that involves either NHEJ or the single-strand annealing recombination pathway; reviewed in 117) or, much less frequently, by maintenance of linear chromosomes through telomere recombination 118. Deletion of *taz1^+^* greatly increases survival with linear chromosomes, which allows easier examination of this pathway, and suggests that Taz1 inhibits recombination at telomeres 118. Rad22 (*S. pombe* Rad52), Tel1, and the MRN complex are required for telomere maintenance in telomerase-negative *taz1*∆ “linear” survivors, suggesting that these cells are similar to *S. cerevisiae* type II survivors 119.

Although Rad52 is a critical protein in the formation of survivors, rare Rad52-independent survivors can arise at a very low frequency in *K. lactis* 113, and in *S. cerevisiae* with long telomeres 120,121. Like type II survivors, these Rad52-independent survivors rely on the amplification of the telomeric repeats. Long telomeres are preferentially elongated in emerging type II survivors 22, so longer telomeres may provide better substrates for recombination, allowing for recombination to happen even in the absence of Rad52. Consistent with this idea, single-strand annealing becomes Rad52-independent when homologous regions are several kilobases long 122, indicating that larger regions of homology can compensate for the lack of Rad52. Another class of Rad52-independent survivors can also occur in cells lacking Exo1 or Sgs1, and thus defective in end-resection 123,124. These survivors have lost telomeric and subtelomeric sequences, but survive by forming large palindromes at chromosome ends.

## RECOMBINATION-MEDIATED TELOMERE MAINTENANCE IN HUMAN CELLS

Although a type I-like ALT cell line has been reported 125,126, most human ALT cancer cells are thought to maintain their telomeres using recombination-mediated mechanisms that resemble what occurs in yeast type II survivors. Much like type II survivors, ALT cells often have long, heterogeneous-sized telomeres 10 127, abundant extrachromosomal DNA circles containing telomeric repeats 59 116 128, and a requirement for the MRN complex and the Sgs1-homolog, BLM, for telomere maintenance 129 130 131. WRN, another Sgs1 homolog, is required for telomere maintenance in some, but not all, ALT cell lines, suggesting the existence of different ALT mechanisms 132. Many ALT cells also possess special promyelocytic leukemia (PML) bodies, termed ALT-associated PML bodies 133 that contain telomeric DNA, shelterin proteins, and DNA damage response and HR factors, including RAD51, RAD52, BLM, WRN, and the MRN complex (reviewed in 134 and 135). Furthermore, ALT cells exhibit an elevated frequency of T-SCEs 136 137.

Interestingly, although several shelterin components have been shown to inhibit telomeric recombination in telomerase-positive mammalian cells (discussed above), the abundance of all six shelterin proteins were unchanged in 22 different ALT cell lines, and exome sequencing failed to detect mutations in any of the genes encoding shelterin proteins 138. The same study found the loss of chromatin-remodeler ATRX in 19 of the 22 cell lines. However, downregulation of ATRX is not sufficient to activate ALT, suggesting that loss of ATRX may only be one step in the process 138. A recent study has found that ATRX represses telomere-bound TERRA in G2/M 139. TERRA, in turn, inhibits hnRNPA1-mediated removal of RPA from telomeres in early S phase, but this inhibition is alleviated in late S phase when TERRA declines at telomeres 140. Thus, ATRX deficiency leads to persistent association of TERRA, and consequently RPA, with telomeres, creating a recombinogenic structure that favors ALT 139. Consistent with this model, TERRA levels are upregulated in ALT cells 138 141. Increased TERRA transcription in *S. cerevisiae* is also thought to promote survivor formation 80, and RNA-DNA hybrids have also been demonstrated to promote the emergence of type II survivors 83.

Another recent study has found that co-depletion of the histone chaperone paralogs ASF1a and ASF1b induces most of the characteristics of ALT cells, including formation of ALT-associated promyelocytic leukemia bodies, presence of extrachromosomal telomeric DNA, increased T-SCEs, and greater telomere length heterogeneity 142. One commonality of ATRX and ASF1 is that they both act on the histone variant H3.3. ATRX, together with DAXX, act together to deposit H3.3 at specific heterochromatic loci, such as telomeres, in a replication-independent manner 143 144 145 146 147. Loss of ATRX or DAXX would impair H3.3 loading at telomeres, and mutations in the genes encoding ATRX, DAXX, and H3.3 are associated with ALT cancers 138,148 149 150. ASF1, originally identified in *S. cerevisiae *151, is a histone chaperone involved in both the replication-coupled and replication-independent incorporation of H3.1-H4 and H3.3-H4 histone dimers into nucleosomes 152,153. It remains to be seen if and how ATRX and ASF1 act together to regulate recombination at telomeres.

## TELOMERE LENGTH AND RECOMBINATION

Short telomeres are more likely to become dysfunctional and may therefore be more susceptible to HR activities. Indeed, analysis of telomere recombination events in telomerase-negative senescing *S. cerevisiae* cells using the telomere sequencing approach described above has revealed a preference for the recombination of short telomeres 21,22,154, and RNA-DNA hybrids formed by TERRA stimulates recombination at short telomeres 81. Short telomeres are also preferentially elongated by recombination in established type II survivors 107,154. In *K. lactis*, short telomeres increase subtelomeric recombination 155, and in senescing cells with a single long telomere, that long telomere almost always serves as the template for BIR initiated by the other shorter telomeres 156.

In contrast, long telomeres are preferentially extended in emerging type II *S. cerevisiae *survivors 22. This finding is consistent with previous studies in prokaryotes, yeast, and mammalian cells showing that the efficiency of HR is proportional to the length of the substrate DNA 157 158 159 160 161 162. The difference in the length preference may be due to which telomeres are in a recombination-competent state. Increasing telomere length may increase the likelihood of recombination, but only if the telomere in question is recombination-competent. In most situations, including in pre-senescent cells and in established survivors, only one or a few short telomeres are in an uncapped state, susceptible to recombination. However, senescent cells primed to become survivors likely have most or all of their telomeres sufficiently eroded and in a recombination-competent state.

## CONCLUDING REMARKS

In this review, we have highlighted the many ways in which HR activities are important at telomeres. However, it is likely that there are additional facets of telomeric HR that have not been explored yet, and much work still needs to be done to determine how these activities are regulated. For example, it is still unclear how some HR activities are suppressed at telomeres (e.g. BIR, extensive end-resection, excision of t-loops, etc.) while others are not (e.g. initial end-resection, t-loop formation, TRD of over-elongated telomeres, etc.). Furthermore, how is the suppression of BIR alleviated in survivor/ALT cells? Considering the importance of HR pathways and telomere biology with respect to cancer and ageing, a better understanding of HR activities at telomeres will have broad ramifications for human health.
